# Glucagon-like peptide 1 receptor agonists in substance use disorders: A systematic review of ClinicalTrials.Gov

**DOI:** 10.1016/j.abrep.2026.100671

**Published:** 2026-01-30

**Authors:** Shruti Patil, Nandini Jha, Manish K. Jha

**Affiliations:** a Medical Student, UT Southwestern Medical Center, 5323 Harry Hines Blvd., Dallas, TX 75390, USA; b Department of Psychiatry, UT Southwestern Medical Center, 5323 Harry Hines Blvd., Dallas, TX 75390, USA; c O’Donnell Brain Institute, UT Southwestern Medical Center, 5323 Harry Hines Blvd., Dallas, TX 75390, USA

## Abstract

**Background:**

Substance use disorders (SUDs) are widely prevalent and associated with high morbidity and mortality. Current treatments have limited efficacy and there are no United States Food and Drug Administration (FDA)-approved treatments for several SUDs (such as methamphetamine, cocaine, and cannabis use disorders). Emerging evidence suggests glucagon-like peptide 1 receptor agonists (GLP-1RAs) may improve outcomes related to SUD. Therefore, a systematic survey of ongoing clinical trials that are evaluating the effects of GLP-1RAs for SUDs is warranted.

**Methods:**

We searched ClinicalTrials.gov from inception to July 2, 2025 (preregistered at: https://osf.io/x58ne/). Inclusion required a GLP-1RA intervention targeting SUDs and outcomes regarding substance use (e.g., urine toxicology, Timeline Follow-Back, craving). Trials excluding individuals with SUDs were excluded.

**Results:**

Of 192 records, 33 met criteria: alcohol use disorder (n = 15), nicotine/tobacco (n = 9), cocaine (n = 4), opioid (n = 4), methamphetamine (n = 1), and none for cannabis. Agents studied included semaglutide (n = 15), exenatide (n = 8), tirzepatide (n = 6), liraglutide (n = 2), dulaglutide (n = 1), and pemvidutide (n = 1). Outcomes and designs were heterogeneous and often mixed self-report with objective indices. Most studies used older GLP-1RAs, focused mainly on alcohol or nicotine/tobacco use disorder, and used a range of outcome measures relying on self-reported and objective measures of substance use.

**Conclusions:**

While GLP-1RAs may represent a paradigm shift for treating SUD, current trials have focused on alcohol and nicotine/tobacco use disorders, with notable gaps for methamphetamine and cannabis use disorders. Trials testing next-generation GLP-1RAs with FDA recommended endpoints are needed to define efficacy and safety across SUDs.

## Highlights

• Thirty-three trials of GLP-1 receptor agonists for substance use disorders identified.
• Most trials are focused on alcohol and nicotine with heterogenous outcomes.
• Few trials for stimulant use disorders, and none for cannabis use disorder.

## 1 Introduction

Substance use disorders (SUDs) are associated with substantial morbidity and mortality due, in part, to overdoses, alcohol related diseases, infectious disease, suicide, and accidental injuries (Perry et al., 2022). Prevalence rates of certain SUDs, such as methamphetamine use disorder, have increased markedly in the past decade which is further complicated by polysubstance use and contamination of supplies with fentanyl (Han et al., 2021). While several pharmacologic agents have been approved by the United States (US) Food and Drug Administration (FDA) for alcohol use disorder (AUD; naltrexone, acamprosate, disulfiram) (McPheeters et al., 2023), tobacco/nicotine use disorder (nicotine replacement therapy, varenicline, bupropion) (Selby & Zawertailo, 2022), and opioid use disorder (OUD; methadone, buprenorphine) (Bell & Strang, 2020), there are no FDA approved treatments for methamphetamine, cocaine, and cannabis use disorder (Clinical Guideline Committee (CGC) Members, 2024; Nielsen et al., 2019). Therefore, there is a significant unmet need for developing novel treatments for SUDs.

Glucagon-like peptide-1 receptor (GLP-1R) agonists (GLP-1RAs), first approved by the FDA in 2005 for the treatment of type II diabetes and recently for chronic weight management, have gained attention for their potential role in the treatment of SUD. These medications activate GLP-1R throughout the pancreas and several other tissues and exert systemic effects, including those in the central nervous system (Collins & Costello, 2025). Furthermore, the GLP-1Rs are expressed in reward-related brain regions (Bruns Vi et al., 2024) and a recent study of individuals with obesity found changes in reward-related brain regions in response to food cues with the use of GLP-1RAs (Martin et al., 2025). In preclinical studies, use of GLP-1RAs is associated with reduced intake and reinforcing properties of addictive substances (Edvardsson et al., 2025, Marquez-Meneses et al., 2025); thereby, supporting their therapeutic potential for SUDs (Volkow & Xu, 2025).

Recent reports based on data from electronic health records provide further evidence for potential therapeutic utility of GLP-1RAs for SUD. Specifically, patients with diabetes who are treated with GLP-1RAs experience better SUD-related outcomes compared to those treated with other antihyperglycemic drugs. A nationwide US Department of Veterans Affairs cohort study found reduced risk of substance use and psychotic disorders with GLP-1RA initiation compared to non-GLP-1RA antihyperglycemics (Xie et al., 2025). Another study across 136 US healthcare systems found patients with OUD or AUD who received GLP-1RAs had significantly lower rates of opioid overdose and alcohol intoxication, and these protective effects were consistent in subgroups with comorbid type 2 diabetes and obesity (Qeadan et al., 2025). Another retrospective cohort study in individuals with obesity found that semaglutide, compared to non-GLP-1RA anti-obesity medications, was associated with lower risk of incident cannabis use disorder in patients with no prior diagnosis, and recurrent diagnosis in patients with history of cannabis use disorder (Wang et al., 2024).

While there have been recent reports reviewing publications based on preclinical studies, clinical trials, and electronic health records data on the effects of GLP-1RAs for SUDs (Bruns Vi et al., 2024, Shen et al., 2024, Sinha and Ghosal, 2025, Yammine et al., 2025), there is still a need to understand the landscape of ongoing clinical trials of GLP-1RAs for SUDs which have been registered but may not be published yet. This is driven in part by the robust pipeline of medications in development for metabolic disorders that target other peptide hormone receptors in addition to GLP-1R (Alfaris et al., 2024, Dos Santos Barbosa et al., 2025), including glucose-dependent insulinotropic peptide receptor (GIPR) agonist such as brenipatide (NCT07223840); glucagon receptor agonist such as pemvidutide (Noureddin et al., 2025) and mazdutide (Ji et al., 2025); amylin receptor agonist such as CagriSema (Davies et al., 2025, Garvey et al., 2025)); glucagon and GIP receptors such as retatrutide, a triple agonist, (Jastreboff et al., 2023); and GIPR antagonist such as maridebart cafraglutide which is administered as monthly injection (Jastreboff et al., 2025). Additionally, orforglipron, a novel small molecule non-peptide GLP-1RA may also be promising (Rosenstock et al., 2025, Wharton et al., 2025, Wharton et al., 2023) given its oral route of administration and similar effects in the brain to the injected peptide GLP-1RAs (Sloop et al., 2024). Therefore, characterizing the ongoing and upcoming clinical trials of GLP-1RAs may inform the therapeutic pipeline of repurposing/developing these medications for SUDs.

The rationale for studying dual/triple agonists (i.e., mediations that target receptors for peptide hormones in addition to GLP-1R) is based in part on the superior metabolic effects of the only FDA approved dual agonist (tirzepatide; dual GLP-1R/GIPR agonist) as compared to GLP-1R-only agonist (Rodriguez et al., 2024). A recent randomized controlled trial (RCT) found greater weight loss and reduction in waist circumference with tirzepatide (10–15 mg/week) versus semaglutide (1.7–2.4 mg/week) (Aronne et al., 2025). Tirzepatide was also more effective than other FDA-approved GLP-1RAs in reducing levels of glycated hemoglobin in RCTs of adults with type 2 diabetes (versus dulaglutide 1.5 mg (Frias et al., 2018) and 0.75 mg/week (Inagaki et al., 2022), and semaglutide 1 mg/week (Frías et al., 2021)). Preclinical models of neurodegeneration suggest that dual GLP-1R/GIPR agonists may be more effective than GLP-1R-only agonists (Maskery et al., 2020, Yuan et al., 2017). However, similar preclinical evidence for SUD is lacking and it remains unclear whether this dual GLP-1R/GIPR agonist property confers greater efficacy (versus GLP-1R-only agonists) for the treatment of SUDs. The greater weight loss-promoting effects of dual/triple agonists (versus GLP-1R-only agonists) may be a potential concern for individuals with SUD using stimulants (such as those with stimulant use disorder) given that use of stimulants can be associated with appetite suppression/weight loss (Jeffers and Benotsch, 2014, Lv et al., 2016).

Therefore, given this active research area of upcoming and planned trials, we undertook a systematic review of clinicaltrials.gov to supplement the knowledge from recently completed systematic review of published studies (Bruns Vi et al., 2024, Shen et al., 2024, Sinha and Ghosal, 2025, Yammine et al., 2025), and focused on key aspects of trials that were registered in clinicaltrials.gov, such as the specific SUDs being studied, specification of eligibility criteria regarding SUD and body mass index, the duration of treatment, the primary and secondary outcome measures, and the sample sizes.

## 2 Methods

This systematic review was conducted in accordance with the Preferred Reporting Items for Systematic Reviews and Meta Analysis (PRISMA) standards. A comprehensive search for clinical trials involving glucagon-like peptide-1 receptor agonists (GLP-1RAs) for substance use disorders (SUDs) was conducted on July 3, 2025 using the ClinicalTrials.Gov database. This is registered as open registration on OSF (preregistered at: https://osf.io/x58ne/) as this is an active research area with potential of updating our research results in a future publication.

ClinicalTrials.Gov was searched to identify trials using GLP-1RAs to treat SUDs from inception until July 2, 2025 using root search terms: “GLP-1”, “Semaglutide”, “Exenatide”, “Tirzepatide”, “Liraglutide”, “Dulaglutide”, and “Lisexenatide”. Each root search term was combined with substance search terms including: “nicotine”, “smoking”, “alcohol”, “cannabis”, “marijuana”, “methamphetamines”, “amphetamines”, “cocaine”, “crack”, “stimulant”, “opioid”, “heroin”, and “fentanyl.” Two authors conducted these searches independently and the senior author then reviewed all the search terms.

Trials were first screened by title and study overview, then inclusion criteria. Trials were included if they (a) had endpoints measuring substance use (i.e., proportion of negative UDS, Timeline Followback, self-reported craving), and (b) utilized a GLP-1RA for the treatment of SUDs (opioid use disorder, alcohol use disorder, methamphetamine use disorder, cocaine use disorder, nicotine/tobacco use disorder, and cannabis use disorder) with or without comorbid conditions (i.e., obesity, type 2 diabetes). Trials were excluded if they excluded individuals with SUDs.

## 3 Results

This systematic review of ClinicalTrials.Gov identified 192 trials via search term and title criteria, with 33 trials satisfying inclusion criteria. The most common reasons for exclusion from this review included the use of glucagon-like peptide-1 receptor agonists (GLP-1RAs) for a medical condition other than substance use disorder (SUD) (e.g., osteoarthritis, diabetes, obesity, cardiovascular disease) and absence of a SUD and/or GLP-1RA treatment group. Included studies were most numerous for alcohol use disorder (N = 15) followed by tobacco/nicotine use disorder (N = 9), cocaine use disorder (N = 4), opioid use disorder (N = 4), and methamphetamine use disorder (N = 1). There were no clinical trials of GLP-1RAs for cannabis use disorder. Semaglutide was the most studied GLP-1RA (including one study in combination with cagrilintide) (N = 15), followed by exenatide (N = 8), tirzepatide (N = 6), liraglutide (N = 2), dulaglutide (N = 1), and pemvidutide (N = 1).

Of the included studies, nine studies are located outside of the United States, see Table 1, Table 2. Thresholds of body mass index (BMI) for study inclusion were highly variable, with some studies excluding underweight or normal weight individuals. Duration of study was also highly variable with durations ranging from 5 days to 32 weeks. Diagnosis of SUD were typically based on structured diagnostic measures and Diagnostic and Statistical Manual Fifth Edition (DSM-5) criteria (American Psychiatric Association (APA), 2013). Primary outcomes focused on reduction of substance use, however, studies varied in how they measured this reduction with some studies focusing on clinical measures (such as the Fagerstrom test for nicotine dependence, cue craving visual analog score, Timeline Followback) and others including biologic data (i.e., Phosphatidylethanol values, urine drug screen).

**Table 1:** Clinical trials of glucagon-like peptide 1 receptor agonists (GLP-1RAs) for alcohol use disorder (AUD).

Trial ID	Substance use related eligibility criteria	BMI Cutoff	Study Status	Estimated start and end dates	Target enrollment number	Interventions	Comparator	Primary outcome	Study Duration	Key secondary outcomes
NCT05895643	Alcohol dependence per ICD-10, AUD per DSM-5. AUDIT > 15. Heavy alcohol drinking defined as more than 6 days with alcohol consumption over 4 units (48 g alcohol) for women and 5 units (60 g alcohol) for men during a consecutive 30-day period, within 40 days prior to baseline evaluation.	Inclusion: ≥30 kg/m^2^	Active, not recruiting	6/13/2023–9/2025	108	Semaglutide	Placebo injection	Change in heavy drinking days	26 weeks	Change in heavy drinking days adjusted for semaglutide dose and weight loss. Total alcohol consumption. Drinks per day. Days without alcohol consumption. Time to relapse. Time to relapse (heavy drinking day). WHO risk levels of alcohol consumption. PACS score. AUDIT score.
NCT05520775	AUD per DSM-5 and NIAAA criteria for current at-risk drinking (i.e., >7/14 drinks in one week for women/men, with at least two episodes of 4+/5 + drinks in the past 30 days)	None	Completed	9/2/2022–4/19/2024	48	Semaglutide	Placebo injection	Change in alcohol consumed and in BAC.	10 weeks	Change in subjective stimulation and sedation on Biphasic Alcohol Effects Scale. Change in Alcohol Demand on Alcohol Purchase Task. Change in Cigarette Demand on Cigarette Purchase Task). Change in daily alcohol and daily cigarette use.
NCT03645408	Exceeds safe weekly drinking limits (4 SDUs for women or 21 SDUs for men per week). At least one episode of binge drinking (>3 SDUs for women, >4 SDUs for men) per week in the four weeks prior to baseline screening. AUD per DSM-5.	Exclusion: <18 or ≥ 30 kg/m^2^.	Terminated	5/2/2019–7/1/2021	8	Exenatide then placebo	Placebo then exenatide	Alcohol consumption	N/A	N/A
NCT06546384	AUDIT-C ≥ 4 for women and ≥ 5 for men.	Inclusion: ≥35 or ≥ 28 kg/m^2^ with weight-related co-morbidities.	Not yet recruiting	6/1/2025–4/30/2027	64	Semaglutide	Behavioral: Weight reduction recommendations	Proportion achieving negative PEth test	16 weeks	Proportion achieving total alcohol abstinence (per TLFB), maintaining total alcohol abstinence (per negative ethylglucoronide test). Change in alcohol abstinent days (per TLFB), heavy drinking days (per TLFB), total number of drinks per week (per TLFB). Effects on reward and relief drinking behavior.
NCT06015893	AUD per DSM-5, >7 drinks/week for females or > 14 drinks/week for males during the 28-day period prior to screening plus at least four days with > 3 drinks for females or > 4 drinks for males during the 28-day period prior to screening, Most recent CIWA-Ar score < 10	Exclusion: <25 or ≥ 50 kg/m^2^	Recruiting	10/17/2023	52	Semaglutide	Behavioral: Take Control + Placebo	Reduction in alcohol drinking. Safety and tolerability of semaglutide.	20 weeks	Reduction in other self-reported alcohol-related outcomes, and blood PEth levels. Reduction in alcohol/food cue-elicited craving assessed in a bar-like laboratory.
NCT05891587	AUD per DSM-5, >7 drinks/week for females or > 14 drinks/week for males during the 28-day period prior to screening plus at least four days with > 3 drinks for females or > 4 drinks for males during the 28-day period prior to screening, Most recent CIWA-Ar score ≤ 10.	Exclusion: <25 or ≥ 50 kg/m^2^	Recruiting	07/07/2023	80	Semaglutide	Placebo injections	Change in alcohol drinking	12 weeks	Change in heavy drinking days, drinks per drinking days, PEth levels, and brain activity in response to alcohol cues during fMRI cue reactivity task. Safety and tolerability of semaglutide.
NCT06994338	Moderate-to-severe AUD, average daily consumption of ≥ 40 g (women) / ≥60 g (men) per day in the 28 days prior to baseline	Inclusion: ≥27 kg/m^2^	Not yet recruiting	06/2025–0/2026	42	Tirzepatide	Placebo	Number of heavy drinking days	8 weeks	N/A
NCT06987513	Moderate-to-severe AUD. Reported ≥ 28 drinks/week if male or ≥ 21 drinks/week if female in the 28 days prior to informed consent; including at least 3 heavy drinking days/week (defined as ≥ 5 drinks/day for male and ≥ 4 drinks/day for female)	Inclusion: ≥25 kg/m^2^	Recruiting	5/15/2025–9/30/2026	100	Pemvidutide 2.4 mg	Placebo	Change in the average number of heavy drinking days/week	24 weeks	Proportion of subjects achieving a 2-level reduction in WHO risk drinking level (per TLFB) during Weeks 21–24. Change in PEth levels at Week 24
NCT03232112	Alcohol dependence per ICD-10. AUDIT score > 15. Heavy alcohol drinking defined as having alcohol consumption over 60 g of alcohol/day (men) or 48 g of alcohol/day (women) for at least 5 days in the past 30 days.	Inclusion: >18.5 kg/m^2^	Completed	8/7/2017–10/05/2020	152	Exenatide 2 mg	Placebo	Heavy drinking days	26 weeks	Total alcohol consumption, PACS, AUDIT and DUDIT score.
NCT06727331	Admitted for the treatment of alcohol withdrawal. AUD per DSM-5.	Inclusion: >25 kg/m^2^	Not yet recruiting	05/2025–07/2026	20	Tirzepatide	Saline Placebo	Cue-induced cravings for Alcohol.	6 weeks	PAC). Percent days abstinent and heavy drinking days. Drinks per drinking day.
NCT06409130	Patient-reported history of alcohol overuse for ≥ 5 years with an alcohol history of a mean of ≥ 50 g (male) or ≥ 40 g (female) per day for the last year leading up to the time of signing informed consent.	Exclusion: ≤25 kg/m^2^.	Active, not recruiting	5/20/24–1/1/2026	270	NNC0194-0499 + semaglutide + cagrilintide	Cagrilintide placebo + semaglutide placebo, NNC0194-0499 placebo + semaglutide placebo	Change in enhanced liver fibrosis	28 weeks	Change in PEth and change in alcohol amount measured by TLFB.
NCT06939088	Alcohol dependence per ICD-10, and AUD per DSM-5. Heavy Alcohol Consumption: ≥4 heavy drinking days (≥48 g for women and ≥ 60 g for men) within a consecutive 21-day period during the 28 days preceding the baseline evaluation.	Inclusion: ≥23 kg/m^2^	Recruiting	5/5/25–12/31/2028	108	Tirzepatide	Placebo	Change in heavy drinking days	26 weeks	Total alcohol consumption. Days without alcohol consumption. WHO risk levels of alcohol consumption. PACS, AUDIT, FTND and DUDIT score. Drug use frequency. Number of cigarettes smoked/day. Blood PEth levels. fMRI alcohol cue-reactivity.
NCT05892432	Moderate-to-severe AUD per DSM-5 using MINI.	Inclusion: >25 kg/m^2^	Recruiting	1/11/24–6/30/2025	135	Semaglutide 3 mg/day for four weeks and 7 mg/day oral for four weeks, oral	Placebo	Change in cue craving VAS	8 weeks	Number of drinks per day. Percentage of heavy drinking days. Change in alcohol cue-elicited brain activation.
NCT07046819	AUD per MINI; current alcohol per TLFB (>14 standard drinks/week for males and > 7 standard drinks/week for females for the last 8 weeks). Liver steatosis (CAP score > 240)	Inclusion: ≥25 and < 40 kg/m^2^	Not yet recruiting	09/10/2025	120	Tirzepatide	Placebo	Percentage reduction of body weight and CAP score.	12 weeks	Reduction in 1) liver steatosis as measured by MRI spectroscopy, 2) MRI visceral fat, 3) liver enzymes, 4) drinking behaviors/cravings, and 5) WHO risk drinking level
NCT07040592	AUD per DSM-5 and AUDIT-C screen. PEth > 20 ng/ml	Exclusion: <23 kg/m^2^	Not yet recruiting	7/1/25	30	Semaglutide	None	Completion of enrollment and duration of sessions	12 weeks	Change in self-reported alcohol use (number of days of alcohol use and number of drinks per day) and PEth.

*Note.* BMI = body mass index; ICD-10 = International Classification of Diseases 10; DSM-5 = Diagnosis and Statistical Manual Fifth Edition; AUDIT (C) is Alcohol Use Disorder Identification Test (consumption); TLFB = the Timeline Followback procedure; PACS = Penn Alcohol Craving Scale; NIAAA is National Institute on Alcohol Abuse and Alcoholism; SDU = standard drink units; MINI = MINI International Neuropsychiatric Interview; WHO = World Health Organization; BAC = breath alcohol concentration; CIWA-Ar = Clinical Institute Withdrawal Assessment for Alcohol – revised; PEth = Phosphatidylethanol; DUDIT = Drug Use Disorders Identification Test; FTND = Fagerström’s Test for Nicotine Dependence; VAS = visual analog scale; MRI = magnetic resonance imaging; CAP = controlled attenuation parameter; and fMRI = functional MRI.

**Table 2:** Clinical trials of glucagon-like peptide 1 receptor agonists for substance use disorders excluding alcohol use disorder (opioid, nicotine, stimulants, cannabis).

Trial ID	Substance use related eligibility criteria	BMI Cutoff	Study Status	Estimated start and end date	Target enrollment number	Interventions	Comparator	Primary outcome	Study Duration	Key secondary outcomes
NCT06548490	Moderate-to-severe OUD per DSM-5. Receiving outpatient treatment and at least 2 weeks on BUP or 4 weeks on methadone at the time of enrollment. Have at least 1 urine test positive for opioids, and positive self-reporting of opioid use.	Inclusion: >18 kg/m^2^	Recruiting	1/13/25–11/2026	200	Semaglutide	Placebo	Abstinent from illicit and nonprescribed opioids.	13 weeks	Self-reported opioid craving scores. Sustained abstinence from opioid use. Abstinence from stimulants use. Days of stimulant use and opioid use (per TLFB).
NCT06651177	Moderate-to-severe OUD	Inclusion: ≥25 kg/m^2^	Not yet recruiting	9/16/25–2/10/2027	310	Tirzepatide	Placebo	6-month MOUD retention rate, UDS.	6 months	Proportion of illicit opioid-negative UDS and negative UDS for other substances. Substance use days. Opioid craving and withdrawal symptoms. Non-Opioid Drug and Alcohol Craving.
NCT06639464	Severe OUD per DSM-5	Exclusion: <25 mg/kg2	Recruiting	06/2025–03/2027	46	Semaglutide	Placebo	Cue-induced cravings. Relapse.	14 weeks	Visual probe task
NCT04199728	OUD and planning on being enrolled in a residential treatment center for a minimum of 4 weeks	None	Completed	10/18/21–9/13/2023	27	Liraglutide	Placebo	Cue-elicited and ambient craving on VAS.	4 weeks	Change in blood oxygenation level response to visual opioid drug cues in prefrontal cortex using functional near infrared spectroscopy.
NCT06924697	A history of smoking for at least one year. FTND score ≥ 4.	None	Enrolling by Invitation	6/21/25–7/1/2026		Semaglutide	DPP-4i	FTND score	Baseline to 24 weeks	fMRI changes (weeks 0 and 24), clinical laboratory tests (weeks 0, 12, and 24), exhaled carbon monoxide test (weeks 0, 12, and 24),
NCT03712098	Self-report smoking cigarettes (menthol and non-menthol) at least 10 times/day, on average, for the past 6 months.	Inclusion: ≥27 kg/m^2^ with one weight-related comorbidity or ≥ 30 kg/m^2^	Completed	11/29/2018–5/25/2022	40	Smoking cessation counseling and liraglutide	Smoking cessation counseling and placebo	7-day point prevalence smoking abstinence at 12 and 26 weeks post-target quit date.	32 weeks	Body weight at 12 and 26 weeks post-target quit date.
NCT05530577	Smoking 5 + cigarettes/day (on average) over the past year, with no period of abstinence > 90 days. Biochemical verification of smoking status, based on expired CO > 8 ppm at baseline	None	Completed	10/7/2022–5/13/2024	24	Semaglutide	Placebo	Change in nicotine self-administration and reinstatement duration.	10 weeks	Change in daily cigarette smoking
NCT02975297	Have smoked ≥ 10 cigarettes a day for at least one year and provide a breath carbon monoxide (CO) ≥ 10 ppm;	Inclusion: ≥25 kg/m^2^	Completed	07/2016–1/3/2020	84	Exenatide	Placebo + NRT plus counseling	Post-quit craving, withdrawal symptoms, and cue-induced craving.	10 weeks	Number of participants who self-reported abstinence and were biochemically verified as abstinent via expired CO Level of ≤ 5 ppm
NCT06173778	Have been smoking ≥ 5 cigarettes per day for at least 1 year (prior to screening) and provide positive cotinine test.	Inclusion: ≥30 kg/m^2^ or ≥ 27 kg/m^2^ with at least one weight-related comorbidity	Recruiting	4/23/2024–8/1/2024	197	Semaglutide	placebo	Change in percent body weight	28 weeks	No smoking related outcomes
NCT05610800	Have been smoking ≥ 5 cigarettes per day for at least 1 year and provide positive cotinine test	Inclusion: ≥25 kg/m^2^	Recruiting	7/12/2022–1/30/2026	140	Exenatide	Placebo	Abstinence between weeks 11 through 14 and biochemically verified	15 weeks	Weight
NCT06986993	Report daily use of > 2 cigarettes per day (CPD), as this is a sufficient threshold for detecting tobacco use disorder	Inclusion: ≥30 kg/m^2^	Recruiting	7/1/2024–6/30/2028	40	Semaglutide	Placebo	Cigarette smoking	12 weeks	Weight
NCT03204396	Daily smokers who are willing to quit and either ≥ 10 cigarettes/day, FTND score ≥ 5, tobacco-associated disease, or treatment with varenicline.	Inclusion: 18–30 kg/m^2^	Completed	6/26/2017–8/30/2022	256	Dulaglutide	0.5 ml normal saline	Abstinence at week 12	52 weeks	Change in body weight
NCT02690987	Nicotine use disorder or AUD per DSM-5 but who are in early stable abstinence (>6 weeks).	Inclusion: 28.0–50.0 kg/m^2^.	Unknown	08/2015–08/2020	95	Exenatide, desacyl ghrelin	Placebo	fMRI activation during cigarette, alcohol and food picture evaluation task	4 years	Resting-state fMRI measure of limbic network
NCT06252623	Self-reported recent use of smoked or intravenous cocaine verified by a positive urine (≥ 150 ng/mL). Report using cocaine for ≥ 10 years and using ≥ 2 g of cocaine/week	None	Withdrawn	08/01/2024–12/31/2026	0	Exenatide	Placebo	Proportion of up to 10 active cocaine doses. Subjective effects of cocaine or placebo.	6 weeks	Incidence of treatment-emergent adverse events
NCT02302976	DSM-IV criteria for cocaine abuse or dependence. Recent street cocaine use in excess of amounts to be administered in the study,positive UDS for cocaine.	None	Completed	11/2014–08/01/2018	13	Cocaine + Exenatide	Placebo + Exenatide, Placebo + Placebo, Cocaine + Placebo	Mean cocaine inter-infusion interval	5 days	N/A
NCT04941521	CUD per DSM-5, have at least 1 cocaine-positive urine specimen (≥ 150 ng/mL) during intake.	None	Completed	06/24/2021–11/17/2021	3	Exenatide and counseling	N/A	Feasibility, safety, cocaine-positive UDS	6 weeks	Cocaine use on ≥ 50% days of the week. Reduction in craving by Week 6 and proportion of participants who had a decrease in drug demand by week 6.
NCT06691243	Meet criteria for CUD per DSM-5. Used cocaine at least 7 out of the past 14 days.	Inclusion: 20–50 kg/m^2^	Not yet recruiting	07/2025–03/2027	40	Semaglutide	Placebo	Meeting criteria for CUD. Cocaine use ≥ 7 out of 14 days	16 weeks	Reduction in cocaine use frequency, craving, risk taking behavior, and impulsivity.
NCT06745128	Moderate-to-severe MtUD. Self-report methamphetamine use on ≥ 18 days in the 30-day period prior to written informed consent using TLFB.	Inclusion: ≥30 kg/m^2^ or ≥ 27 kg/m2 and at least one weight-related comorbidity	Active, not recruiting	2/3/2025–3/31/2026	45	Tirzepatide	N/A	Self-reported use of methamphetamine (TLFB)	32 weeks	Feasibility of using tirzepatide in individuals with MtUD

*Note*. DSM-5 = Diagnosis and Statistical Manual Fifth edition; OUD = opioid use disorder; BUP = buprenorphine; MOUD = medication for opioid use disorder; UDS = Urine Drug Screen; VAS = visual analog scale; FTND = Fagerström Test for Nicotine Dependence; NRT = nicotine replacement therapy; CO = carbon monoxide; ppm = parts per million; CUD = cocaine use disorder; TLFB = the Timeline Followback procedure; fMRI = functional magnetic resonance imaging; and MtUD = methamphetamine use disorder.

As shown in Table 1, a majority (N = 7) of trials for alcohol use disorder are studying semaglutide, followed by tirzepatide (N = 4), and exenatide (N = 2). One study (NCT05520775) did not have a BMI criterion, while the remaining 14 excluded underweight individuals with variable BMI cutoffs. Five studies also had variable upper limit for BMI criterion. Of the included studies, two (NCT05520775 and NCT03232112) have been completed and one (NCT03645408) was terminated due to hospital-wide policies that halted recruitment due to novel coronavirus 19 (COVID-19). The EXALT trial (NCT03232112) found that although exenatide did not significantly reduce the number of heavy drinking days compared with placebo, it significantly attenuated functional magnetic resonance imaging alcohol cue reactivity in the ventral striatum and septal areas, regions crucial for drug reward and addiction. Furthermore, this study found lower dopamine transporter availability in the exenatide group compared with placebo. In the obese subgroup, exploratory analysis revealed significantly reduced heavy drinking and alcohol intake in the exenatide group compared with placebo (Klausen et al., 2022). The Semaglutide for Alcohol Use Disorder study (NCT05520775) found that low-dose semaglutide had a medium to large effect size for grams of alcohol consumed and peak breath alcohol concentration. This study also found that semaglutide did not affect average drinks per calendar day or number of drinking days, but significantly reduced drinks per drinking day and weekly alcohol craving (Hendershot et al., 2025).

As shown in Table 2, a majority of trials (N = 4) for nicotine/tobacco use disorder utilized semaglutide, followed by exenatide (N = 3). Two studies did not have a BMI criterion (NCT06924697 and NCT05530577), while six trials excluded participants not overweight/obese. The remaining trial excluded obese participants (NCT03204396). Two of four completed studies have published results. The Exenatide Once Weekly for Smoking Cessation study (NCT02975297) found that exenatide was associated with higher likelihood of smoking abstinence compared to placebo, and that exenatide reduced end-of-treatment craving and withdrawal among abstainers (Yammine et al., 2021). The Smoking Cessation Facilitated by Glucagon-like Peptide-1 (GLP-1) Analogues study (NCT03204396) found no significant difference in abstinence rates between the dulaglutide and placebo groups, and that craving for smoking declined during treatment with no difference between dulaglutide and placebo groups (Lengsfeld et al., 2023). For opioid use disorder, semaglutide was the most frequently studied GLP-1RA, see Table 2. There is one completed study for opioid use disorder without published results. For cocaine use disorder, exenatide (N = 3) was the most frequently studied GLP-1RA; however, one study of exenatide for cocaine use disorder was withdrawn due to the inability to acquire the study drug (NCT06252623). One completed study (NCT02302976) for cocaine use disorder did not have published results whereas another was published as a case series to support feasibility of using exenatide 2 mg subcutaneously once-weekly in an open-label fashion for 6 weeks (Yammine et al., 2023). There is one reported study of tirzepatide for methamphetamine use disorder. There are no studies of GLP-1RAs for cannabis use disorder (Table 2).

After the completion of the review for this report, a Phase 3 program was registered in November 2025 for a novel dual GLP-1R/GIPR agonist (brenipatide) which is seeking to enroll individuals with moderate-to-severe alcohol use disorder in two large (n = 1100 each) RCTs (NCT07219966 and NCT07219953). Additionally, a smaller trial (n = 222) is also evaluating the effect of this medication in individuals who recently quit smoking and are motivated to stay quit (NCT07223840).

## 4 Discussion

This systematic review of clinical trials focusing on glucagon-like peptide-1 receptor agonists (GLP-1RAs) for the treatment of substance use disorders (SUDs) has several important findings. Firstly, alcohol use disorder (AUD) was the most extensively studied SUD, followed by nicotine/tobacco use disorder, and cocaine use disorder. There was only one trial for methamphetamine use disorder, and no clinical trials for cannabis use disorder. Semaglutide was the most studied GLP-1RA for AUD and nicotine use disorder, while exenatide was the most studied GLP-1RA for cocaine use disorder. Included studies had a wide range of body mass index (BMI) cutoffs, length of study, methods of measurement of SUD-related outcomes, and primary outcome measures.

Although there are several medication options for both AUD and nicotine/tobacco use disorder, patients with co-morbid obesity or with contraindications to current modalities would likely benefit from the use of GLP-1RA as part of their treatment approach. However, the variability in eligibility criteria based on BMI as a measure of obesity may raise concerns for the therapeutic potential of GLP-1RAs in normal/underweight individuals. Greater access to potential therapeutic options for SUDs (such as GLP-1RAs in primary care settings if shown to be effective in ongoing/future trials) may enable higher treatment initiation and maintenance. Furthermore, with disorders such as cocaine use disorder, methamphetamine use disorder, and cannabis use disorder, there are no Food and Drug Administration (FDA) approved therapies, leaving a gap in care that GLP-1RAs may be able to fill. However, there is a paucity of ongoing/upcoming clinical trials of GLP-1RAs for these disorders despite studies based on electronic health records suggesting their therapeutic potential (Wang et al., 2024, Xie et al., 2025).

The included studies in this review suggest that the overall number of trials are low and there are a wide variety of approaches being used in measuring the utility of GLP-1RAs for the treatment of SUD. The variability in approaches likely reflects the differences in guidance for different SUDs from regulatory agencies like the FDA. However, greater effort is needed for harmonization of endpoints that are consistent with FDA’s guidance within SUD category (FDA, 2020, FDA, 2023, Witkiewitz et al., 2025) and the need to characterize the changes in use of other substances in individuals with polysubstance use.

Existing studies support the link between SUDs and obesity (Bliss and Whiteside, 2018, Brutman et al., 2019), arguing that the similar biological pathways responsible for pathologic consumption of highly palatable food may also responsible for pathologic consumption of other substances (Leggio et al., 2025, Volkow et al., 2013). Therefore, it is encouraging that several ongoing studies are assessing changes in brain activity in response to drug cues as well as food cues after treatment with GLP-1RAs. However, studies are needed to further elucidate the mechanisms by which these relatively large molecules (peptides) affect the function of GLP-1Rs in brain. Further research with GLP-1RAs would also allow for determination of therapeutic dosage and duration, long term efficacy, and safety profile and tolerability within the SUD population. Systematic efforts to identify patient characteristics that are associated with higher likelihood of therapeutic benefits for SUDs are also needed. All of the ongoing studies in SUD have used GLP-1RAs that are injected subcutaneously (except one of oral semaglutide), and future studies are needed with orally available compounds (potentially increase acceptability and access) (Rosenstock et al., 2025, Wharton et al., 2025, Wharton et al., 2023) and with longer duration of action (such as monthly injections which may improve compliance) (Jastreboff et al., 2025).

## 5 Conclusion

Substance use disorders remain prevalent, with high rates of morbidity and mortality, and few therapeutic options. There is a large unmet need especially for substance use disorders like cocaine use disorder, methamphetamine use disorder, and cannabis use disorder with no current Food and Drug Administration (FDA) approved medications. This review of completed and ongoing clinical trials revealed that glucagon-like peptide-1 receptor agonists (GLP-1RAs) are being mostly commonly studied in individuals with alcohol use disorder. While GLP-1RAs may represent a paradigm shift for treating substance use disorders, randomized controlled trials for methamphetamine and cannabis use disorders are lacking. Future trials should utilize harmonized endpoints based on FDA guidance to define efficacy and safety and test next-generation GLP-1RAs, including those available orally and with longer duration of action.

## CRediT authorship contribution statement

**Shruti Patil:** Writing – original draft, Methodology, Investigation, Data curation, Conceptualization. **Nandini Jha:** Writing – review & editing, Methodology, Investigation, Data curation, Conceptualization. **Manish K. Jha:** Writing – review & editing, Supervision, Methodology, Investigation, Funding acquisition.

## Funding

This work was supported by the O’Donnell Brain Institute Clinical Neuroscience Endowed Scholar Award to Manish Jha.

## Declaration of competing interest

The authors declare the following financial interests/personal relationships which may be considered as potential competing interests: In the past 36 months, Dr. Jha has received contract research grants from Neurocrine Bioscience, Navitor/Supernus and Janssen Research & Development; honorarium to serve as Section Editor of the Psychiatry & Behavioral Health Learning Network and as Guest Editor for Psychiatric Clinics of North America from Elsevier; consultant fees from Janssen Scientific Affairs, Sanofi, Neurocrine, AbbVie, MindMed and Boehringer Ingelheim; fees to serve on Data Safety and Monitoring Board for Worldwide Clinical Trials (Eliem, Skye and Inversargo), Vicore Pharma and IQVIA (Click); and honoraria for educational presentations from North American Center for Continuing Medical Education, Medscape/WebMD, Clinical Care Options, Physicians’ Education Resource, Efficient CME, Soterix, and H.C. Wainwright & Co. Others authors have no conflicts of interest to disclose.

## Data Availability

No data was used for the research described in the article.
